# Glutaraldehyde-pea protein grafted polysaccharide matrices for functioning as covalent immobilizers

**DOI:** 10.1038/s41598-023-36045-z

**Published:** 2023-06-05

**Authors:** Marwa I. Wahba

**Affiliations:** 1 Department of Chemistry of Natural and Microbial Products, National Research Centre, El-Behooth St., Dokki, Giza, Egypt; 2 Centre of Scientific Excellence-Group of Advanced Materials and Nanotechnology, National Research Centre, El-Behooth St., Dokki, Giza, Egypt

**Keywords:** Biochemistry, Biotechnology

## Abstract

Three polysaccharide matrices (κ-Carrageenan (Carr), gellan gum, and agar) were grafted via glutaraldehyde (GA) and pea protein (PP). The grafted matrices covalently immobilized β-d-galactosidase (β-GL). Nonetheless, grafted Carr acquired the topmost amount of immobilized β-GL (iβ-GL). Thus, its grafting process was honed via Box-Behnken design and was further characterized via FTIR, EDX, and SEM. The optimal GA-PP-Carr grafting comprised processing Carr beads with 10% PP dispersion of pH 1 and 25% GA solution. The optimal GA-PP-Carr beads acquired 11.44 Ug^−1^ iβ-GL with 45.49% immobilization efficiency. Both free and GA-PP-Carr iβ-GLs manifested their topmost activity at the selfsame temperature and pH. Nonetheless, the β-GL K_m_ and V_max_ values were reduced following immobilization. The GA-PP-Carr iβ-GL manifested good operational stability. Moreover, its storage stability was incremented where 91.74% activity was offered after 35 storage days. The GA-PP-Carr iβ-GL was utilized to degrade lactose in whey permeate with 81.90% lactose degradation efficiency.

## Introduction

Pea protein (PP) constitutes a 20–25% fraction of pea seeds^1^. PP comprises four major constituents: globulins (55–65%), albumins (18–25%), prolamin (4–5%), and glutelin (3–4%)^2^. PP globulins are further subdivided into, chiefly, legumin and vicilin. Minute quantities of convicilin are also present amid PP globulins^1^. PP offers numerous beneficial traits, such as being widely available at reasonable cost. PP is also gluten and allergen-free^3^. PP has been occasionally coupled to polysaccharides in order to enhance and modulate its traits^4^. For instance, the gastric digestion of PP was reduced to ~ 13% after its formulation as PP-ҡ-carrageenan (Carr) capsules^5^. On other occasions, the enhanced nutritive value and amino acid content of PP encouraged its incorporation with variable polysaccharides in food related applications. For instance, PP-gellan gum and PP-agarose blends were investigated as supporting structures for propagating cells during the preparation of cultured meat^6^. PP was also blended with agar so as to function as partial or complete substitutes for fat in meat emulsion models^7^. Nonetheless, PP was never coupled with polysaccharides in order to attain covalent immobilizers.

Immobilization could improve enzyme traits and make them suitable for industrial applications. Immobilization could heighten the stability of enzymes whether they are monomeric or multimeric^8^. Enzymes qualities could also be modified following immobilization, such as their activity, selectivity and specificity. Moreover, immobilization enables the recurrent exploitation of enzymes. Noteworthy, enzymes immobilization via covalent bonds would prohibit their release^9^, and this would help maintain the biocatalysts efficiencies for prolonged intervals.

Polysaccharides, such as Carr, agar, and gellan-gum (GEG), don’t comprise the functional residues which could enable them to covalently bind enzymes^10^. Nonetheless, grafting PP to these polysaccharides would enable their functionalization into covalent immobilizers. PP would bind to these polysaccharides via ionic-exchange, hydrogen-bonding, and hydrophobic interactions owing to PP proteinaceous nature^1^ and also to its reported surface hydrophobicity^11^. After binding to the polysaccharides, PP would present nucleophilic amine moieties that would react with glutaraldehyde (GA)^12^. GA would then covalently link enzymes. Accordingly, Carr, GEG, and agar were grafted via PP and GA in order to acquire covalent immobilizers.β-D-Galactosidase (β-GL) was adopted during our study. β-GL hydrolyzes the β-1,4-glycoside linkages established amidst galactose and variable carbohydrate entities. Such linkages are present in lactose^13^. Lactose degradation via β-GL would provide the low lactose or the lactose free dairy products which are crucial for lactose-intolerant people. Reducing lactose would also reduce crystallization, and this would boost the smoothness of variable dairy products, such as cheese, curd, and ice-cream^14^. Whey, a byproduct of dairy industries, could also be subjected to β-GL mediated lactose degradation. Such degradation would valorize whey and encourage its exploitation as it would boost its sweetness and promote its exploitation as sweet syrup in variable food industries^15^.

Since immobilization is needed to improve enzymes traits and make them suitable for industrial applications^8^, there is a constant need to prepare new immobilizers. These immobilizers should be efficient and also cost-effective. Thus, in this research, the abundant, cost-effective PP was exploited, in a novel manner, in order to prepare three new immobilizers. Carr, agar, and GEG were converted into covalent immobilizers following a GA-PP grafting protocol. The GA-PP-Carr was the most proficient immobilizer which acquired the topmost amount of immobilized β-GL (iβ-GL). Thus, its grafting process was further improved via Box-Behnken design. Various stability studies were also conducted on the GA-PP-Carr iβ-GL in order to verify its suitability for industrial applications. Finally, the GA-PP-Carr iβ-GL was inspected in one of its potential applications which involved whey permeate lactose degradation.

## Materials and methods

### Materials

Carr (Gelcarin GP 812®) was acquired from PhytoTechnology Laboratories®, USA (lot number: 01F25764D). Agar with 5% sulfated ash was the product of SD Fine chemicals, India (batch number: 106Y/1106/1309/61). GEG (kelcogel® F) was acquired from Modernist Pantry, USA. Noteworthy, the molecular weight of kelcogel® F was reported to be 250 kDa^16^. *Aspergillus oryzae* β-GL, 50% GA solution, and KCl were bought from Sigma, Germany. PP (81.25% protein) was acquired from 138 Foods, Inc., USA.

### Methods

#### Preparation and grafting of the variable polysaccharide matrices

Agar (5%)^17^ and GEG (3%)^15^ solutions were obtained through the stirring of the respective polysaccharide powder in distilled water within a boiling water-bath. In case of agar, ~ 25 g of the hot agar solution was then transferred to a 9 cm plastic petri-dish, and was left aside till it solidified. Afterwards, the attained thick agar film was cut into small disks with a cork–borer^17^. As regards to GEG, a thin syringe needle was exploited to drip the hot GEG solution into 5% CaCl_2_·2H_2_O solution. The attained GEG beads were kept in the CaCl_2_ gelling solution for at least 18 h before scrupulously washing them^15^. In case of Carr, a 2% Carr solution was obtained after stirring the Carr powder in distilled water within a 70 °C water-bath. A thin syringe needle was then exploited to drip the hot Carr solution into 3% KCl solution^18^. The attained Carr beads were kept in the gelling KCl solution for at least 18 h before scrupulously washing them.

Afterwards, ~ 2 g of the agar disks or the washed GEG or Carr beads were individually mixed with ~ 10 ml PP suspension [10% (w/w); pH 1]. Such mixtures were rotated overnight on a roller-stirrer. The disks or beads were then scrupulously washed until all the unbound PP was removed. Afterwards, GA solution (4 ml; 5% v/v) was individually added to each of the polysaccharide matrices for 1 h. Finally, the GA-PP grafted polysaccharide matrices were scrupulously washed and were exploited to load β-GL. Noteworthy, the GA was diluted (v/v) from the commercial 50% GA solution.

It should be noted that when the GEG and agar grafting processes were optimized with respect to the PP pH, the aforementioned procedure was adopted but the PP pH was altered amid a 1–9 pH range. On the other hand, when the GA-PP Carr grafting was honed via Box-Behnken design (BBD), the PP pH, PP concentration, and GA concentration were altered as per the BBD. Design Expert 13 was exploited to analyze the BBD data.

#### β-GL immobilization

This was accomplished as a modification to the formerly adopted β-GL immobilization protocol^15,18^. In brief, the GA-PP grafted polysaccharide matrices (0.3 g) were individually mixed with 3 ml β-GL solution, which was constituted within 0.3 M citrate–phosphate (pH 4.9). Such mixtures were rotated overnight on a roller-stirrer. The β-GL loaded GA-PP grafted polysaccharide matrices were then scrupulously washed to remove any unbound β-GL and their activity was estimated.

### Estimation of β-GL activity

Both the iβ-GL and its free compeer were assayed. In case of the iβ-GL, ~ 0.06 g of the GA-PP grafted polysaccharide matrices, which were loaded with β-GL, were placed within 0.5 ml buffer (0.1 M citrate–phosphate, pH 4.9). At the same time, the free β-GL powder was separately dissolved in 0.5 ml of the selfsame buffer. The substrate solution was then prepared, and it comprised a 200 mM lactose solution in citrate–phosphate buffer (pH 4.9; 0.1 M). The enzymatic reaction was initiated upon combining the 0.5 ml buffer, which comprised either free or iβ-GL, with 3.5 ml lactose solution. This reaction continued for 15 min within a 37 °C shaking-water-bath. A part of the reaction solution was then taken and put in a boiling-water-bath (~ 10 min). Afterwards, the reaction solution was cooled to room temperature, and its glucose content was investigated via commercial glucose kits. These kits utilized glucose oxidase to estimate glucose as per the Trinder method^19^.One β-GL unit (U) corresponded to the release of 1 μmol glucose/min whilst adopting the aforementioned enzymatic reaction.

#### Characterization

##### FTIR analysis

The Carr beads, the optimal PP-Carr beads, and the optimal GA-PP-Carr beads were crushed while wet. Afterwards, they were dried in a 50 °C oven and they were analyzed by FT-IR spectrometer 4100 JASCO, Japan, as per the KBr pellet method^20^. In brief, the powdered sample was blended with solid KBr and was compressed into a pellet. The spectrum of the sample pellet was then recorded amid 4000–400 cm^−1^ versus neat KBr pellet (blank).

##### Elemental construction

The Carr beads, the optimal PP-Carr beads, and the optimal GA-PP-Carr beads were lyophilized. The weight percents of their constituent elements were then inspected, without any further processing, via Energy-dispersive X-ray (EDX) analysis. This analysis was performed by the Octane pro, AMETEK® EDAX, USA. In all specimens, only the carbon, nitrogen, oxygen, and sulfur were investigated. However, chloride and potassium were also investigated in the Carr beads owing to their bountiful presence.

##### Scan electron microscope (SEM) inspection

The Carr beads, the optimal PP-Carr beads, and the optimal GA-PP-Carr beads were lyophilized. Afterwards, their surfaces were inspected, without any further processing, via the SEM Quanta 250 FEG, The Netherlands.

#### Temperature profile and reckoning of activation energy

The β-GL enzyme assay was accomplished. Nonetheless, the water-bath temperature was varied (37–65 °C). The topmost recorded activity was denoted as the 100%, and the rest of the recorded activities were presented in proportion to this 100% activity. Moreover, the activities recorded at 37–56 °C were reckoned as initial lactose degradation rates (V_0_; ML^−1^ s^−1^). A plot of ln V_0_ vs 1/temperature (Kelvin) was then constructed, and its slope (− E_a_/R) was exploited to reckon the activation energy of lactose degradation (E_a_)^18^. R represented the universal gas constant (8.314 JK^−1^ mol^−1^).

#### Inspection of free and GA-PP-Carr iβ-GLs pH profiles

The β-GL enzyme assay was accomplished. Nonetheless, the buffer pH was varied (1.7–7.3). The topmost recorded activity was denoted as the 100%, and the rest of the recorded activities were presented in proportion to this 100% activity.

#### Reckoning of K_m_ and V_max_

The β-GL enzyme assay was accomplished. Nonetheless, the substrate concentration was varied amid 50–200 mM concentration. Thus, the final substrate concentrations ranged from 43.75 to 175 mM. The activities acquired via both the free and the GA-PP-Carr iβ-GLs were then presented per mg of solid β-GL. The Hanes-Woolf plot was drawn and its straight line equation was exploited to reckon the V_max_ (1/slope) and K_m_ (Y – intercept × V_max_).

#### Reusability

The activity of a portion of the β-GL loaded GA-PP-Carr beads was estimated and denoted as 100%. Afterwards, the selfsame beads were washed twice with the 0.1 M citrate–phosphate buffer (pH 4.9). Their activity was then re-estimated and presented in proportion to the initial 100%.

#### Storage stability

The GA-PP-Carr beads were loaded with β-GL. The activity of a portion of these beads was estimated and denoted as 100%. The rest of the loaded beads were kept in the fridge in distilled water. After predetermined durations, the activity of a new portion of the stored beads was estimated and presented in proportion to the initial 100%.

#### Stability in presence of Ca^2+^, Al^3+^, SDS, and EDTA

In case of the iβ-GL, ~ 0.06 g of the GA-PP-Carr beads which were loaded with β-GL, were placed within 0.25 ml buffer (0.1 M citrate–phosphate, pH 4.9). Similarly, the free β-GL powder was dissolved in 0.25 ml of the selfsame buffer. Meanwhile, 20 mM solutions of the additives (CaCl_2_, AlCl_3_, sodium dodecyl sulfate (SDS), and ethylenediaminetetraacetic acid (EDTA)) were individually prepared in the selfsame citrate–phosphate buffer. These additives solutions (0.25 ml) were then added to both enzymes specimens so that the final additives concentrations were 10 mM. The mixtures were left aside at room temperature for 1 h. Afterwards, 3.5 ml lactose solution was added to the mixtures and the β-GL activity assay was conducted as mentioned above. The activities of the mixtures were presented relative to the activity of their control. In the control, 0.25 ml buffer was mixed with the enzyme specimen instead of the additive solution.

#### Whey permeate (WP) lactose degradation

WP was brought from a local cheese factory. It was placed in a boiling water-bath for 1 h. After cooling, the WP was filtered in order to discard the precipitated proteins, and its pH was set at 5.38. The WP was filtered once more before utilizing it. The lactose content of the WP was reckoned as per Nickerson et al.^21^. In brief, 8 ml WP was blended with 1 ml distilled water and 1 ml ZAPT reagent (zinc acetate (5 g), phosphotungestic acid (2.5 g), and glacial acetic acid (4 ml) made up to 20 ml with distilled water). This blend was left on bench for 10 min and then it was filtered. The filtrate (0.5 ml) was blended with 0.5 ml 1N NaOH and 9 ml distilled water and was filtered again. Meanwhile, 100 ml glycine–NaOH buffer was prepared by blending 0.22 M glycine-0.32 M NaCl solution (15 ml) with 0.385 M NaOH solution (85 ml). Afterwards, 0.8 ml of the filtrate was blended with 0.8 ml buffer, 80 µl methylamine-HCl aqueous solution (5%), and 80 µl sodium sulfite aqueous solution (1%). This blend was incubated within a 75 °C water-bath and after 25 min its O.D. was measured at 540 nm. Noteworthy, the WP lactose content was found to be 64.05 ± 2.54 µmol/ml.

In order to accomplish the WP lactose degradation, 1.16 U of the GA-PP-Carr iβ-GL was mixed with 5 ml WP. The mixture was placed in a 50 °C shaking-water-bath. After predetermined durations, 20 µl of the WP was pipetted, diluted, and assayed for its glucose content. After accomplishing the first lactose degradation cycle, which lasted 39 h, the selfsame β-GL loaded GA-PP-Carr beads were scrupulously washed with distilled water, and were mixed with 5 ml fresh WP. Three reusability cycles were accomplished and each lasted 24 h. Noteworthy, lactose degradation efficiency (LDE) was reckoned as follows:

$${\text{LDE }}\left( \% \right) \, = {\text{ Amount}}\;{\text{ of }}\;{\text{released }}\;{\text{glucose }}(\mu {\text{mol}}/{\text{ml}})/{\text{inceptive }}\;{\text{WP }}\;{\text{lactose }}\;{\text{content }}(\mu {\text{mol}}/{\text{ml}})*{1}00.$$

#### Statistics

The BBD was analyzed via Design Expert 13. Elsewhere, Excel One Way ANOVA together with Tuckey Kramer post hoc were utilized to assess the significant differences amid the results (P-value < 0.05). However, if only two sets of results were compared the Excel two-sample t-test was adopted.

## Results and discussion

### Screening of variable biopolymers

**Figure 1:**
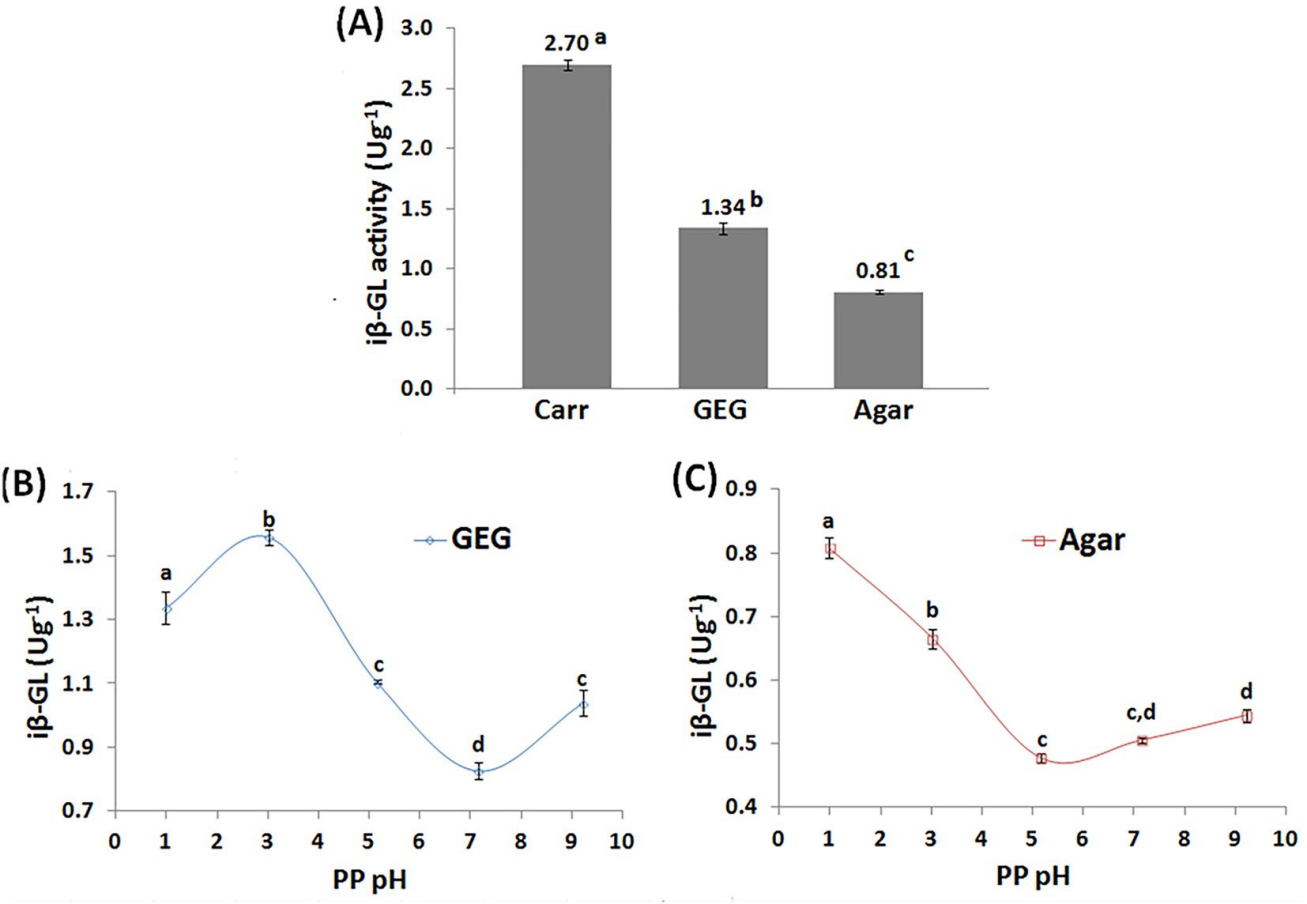
(**A**) Amounts of iβ-GL activities acquired via the GA-PP grafted agar, Carr and GEG matrices. Effect of altering the PP pH on the iβ-GL activities acquired via the GA-PP grafted (**B**) GEG and (**C**) agar matrices (Variable low case letters reflected significant difference amid the results).

Three biopolymers were grafted via PP and GA in order to acquire β-GL covalent immobilizers. These were: k-carrageenan (Carr), gellan-gum (GEG), and agar. The amount of β-GL immobilized via grafted Carr was 2.02 fold larger than that immobilized via grafted GEG. Moreover, it was 3.34 fold larger than that immobilized via grafted agar (Fig. 1A). Thus, the GA-PP Carr grafting process was selected to be thoroughly honed via BBD and to be further characterized and investigated. As regards to the grafting processes of GEG and agar, only one factor was investigated and it was selected to be the PP pH. Protein pH was the most significant factor that influenced Carr grafting via GA and whey protein isolate as indicated by the ANOVA of the BBD^22^. Moreover, Protein pH was the most influential factor that affected the GA-soy protein isolate grafting of Carr^18^.

### Effect of PP pH on the grafting of GEG and agar

The optimum PP pH, which enabled the acquisition of the topmost amount of iβ-GL, was pH 1 in case of agar and pH 3 in case of GEG (Fig. 1B,C). On another occasion, when PP was utilized to construct coacervates with variable polysaccharides, the pH optima were also varied upon altering the adopted polysaccharide^3,23^. Complex coacervation depends on the ionic-exchange amid two biopolymers that exhibit opposite charges^24^. Thus, it holds some similarity to the PP-polysaccharide grafting process proposed herein. It was stated that the topmost quantity of PP-gum-tragacanth coacervates was acquired at an optimum pH of 4.49 when the coacervates were investigated at a ratio of 1:1^3^. On the other hand, the optimum pH was 2.1 when PP and alginate coacervates were investigated at a 1:1 ratio^23^. It was also reported that the optimum pH for complex coacervation amid PP and gum-arabic (ratio 1:1) was ~ 3^3^. This PP-gum-arabic optimum pH was analogous to the optimum pH reported herein for GEG grafting. Such similarity could be regarded to the anionic glucuronic acid moieties which are constituents of both GEG^10^ and gum-arabic^3^. It should also be noted that when GEG was grafted via GA and egg-white protein, the topmost amount of iβ-GL was acquired at an egg-white protein pH of 3^15^.

In order to rationalize the variable pH optima recorded herein during agar and GEG grafting, the charges presented via PP, agar and GEG should be investigated. These charges govern the ionic-exchange amid PP and agar or GEG, and their variance would alter such ionic-exchange. Ionic-exchange was reported to mediate the interactions amid variable proteins and polysaccharides^3,4,15,22^. GEG (pKa 3.5) comprises glucuronic acid moieties. Furthermore, it was proven that GEG (kelcogel® F) manifested negative zeta potential amid 2.7–7 pH range. Nonetheless, its electro-negative traits were progressively incremented as the pH was raised from 2.7 to 4. Further raising the pH from 4 to 7 didn’t significantly alter GEG zeta potential and it was nearly fixed at – 31 mV^16^. From this pattern, it could be implied that more anionic moieties would be presented by GEG at pH 3 than at pH 1. The GEG lower content of anionic moieties at pH 1 could also be verified from Henderson-Hasselbach equation. According to this equation the ionized acid residues that would be presented by GEG (pKa 3.5^16^) at pH 1 would constitute only 0.32% of its total acid residues. The larger quantity of anionic moieties that would be presented by GEG at pH 3 would promote its ionic-exchange with the cationic PP (pI 4.5–4.8^3,4^). This would eventually create of a finer GEG immobilizer and would raise the amount of iβ-GL at pH 3. At pH 5 GEG would be more electronegative than at pH 3^16^ and a finer ionic-exchange would be expected. Nonetheless, PP (pI 4.5–4.8^3,4^) would be close to its pI. Putting PP nearer to its pI was previously reported to be coupled with an increase in aggregation amid its entities^3,23^. Increased aggregation amid PP would affect the amount of its exposed charged amine residues^23^. If these exposed charged amine residues were reduced, the ionic-exchange amid PP and the anionic GEG would be impaired. Moreover, the cationic moieties presented by PP (pI 4.5–4.8^3,4^) at pH 5 would be somewhat lesser than its presented anionic moieties. Lowering the amount of the cationic moieties presented by PP and increasing the aggregation amid PP moieties would impair its ionic-excahnge with GEG, and this would eventually reduce the amount of iβ-GL. Thus, the optimal PP pH for GEG grafting was pH 3 rather than pH 5.

Noteworthy, agar zeta potential was formerly explored amid a 2–10 pH range, and it was shown to be nearly constant at − 20 mV along the entire tested pH range^25^. Despite such constant zeta potential, significant alterations were recorded in the amount of the grafted agar iβ-GL when the pH was altered amid 3–9 (Fig. 1C). Thus, the alterations in the iβ-GL would be mainly regarded to PP rather than to agar. PP zeta-potential was shown to be significantly incremented upon gradually lowering its pH from its pI (4.5–4.8) to pH 2 (lowest explored pH value)^3,4^. Thus, it could be expected that PP would be more cationic at pH 1 than at pH 3. The incremented amounts of PP cationic moieties, which would be presented at pH 1, would establish a finer ionic-exchange with agar. Thus, the topmost amount of grafted agar iβ-GL was obtainable at pH 1. It should be noted that agar would exhibit good anionic traits at pH 1 as it comprises anionic sulfate moieties^17^ analogous to those presented by Carr. Carr sulfate moieties were previously reported to exhibit pKa of ~ 2^26^.

### Optimization of the GA-PP grafting of Carr beads

1 $${\text{i}}\beta {\text{ - GL }} = { 1}.{43}00 \, {-} \, 0.{\text{7663 A }}{-} \, 0.{\text{2494 B }} + \, 0.{44}0{\text{6 C }}{-} \, 0.{\text{1912 AB }}{-} \, 0.{13}0{\text{3 AC }}{-} \, 0.{23}0{\text{6 BC }} + { 1}.{88}00{\text{ A}}^{{2}} + \, 0.0{\text{928 B}}^{{2}} + \, 0.0{\text{325 C}}^{{2}}$$

**Figure 2:**
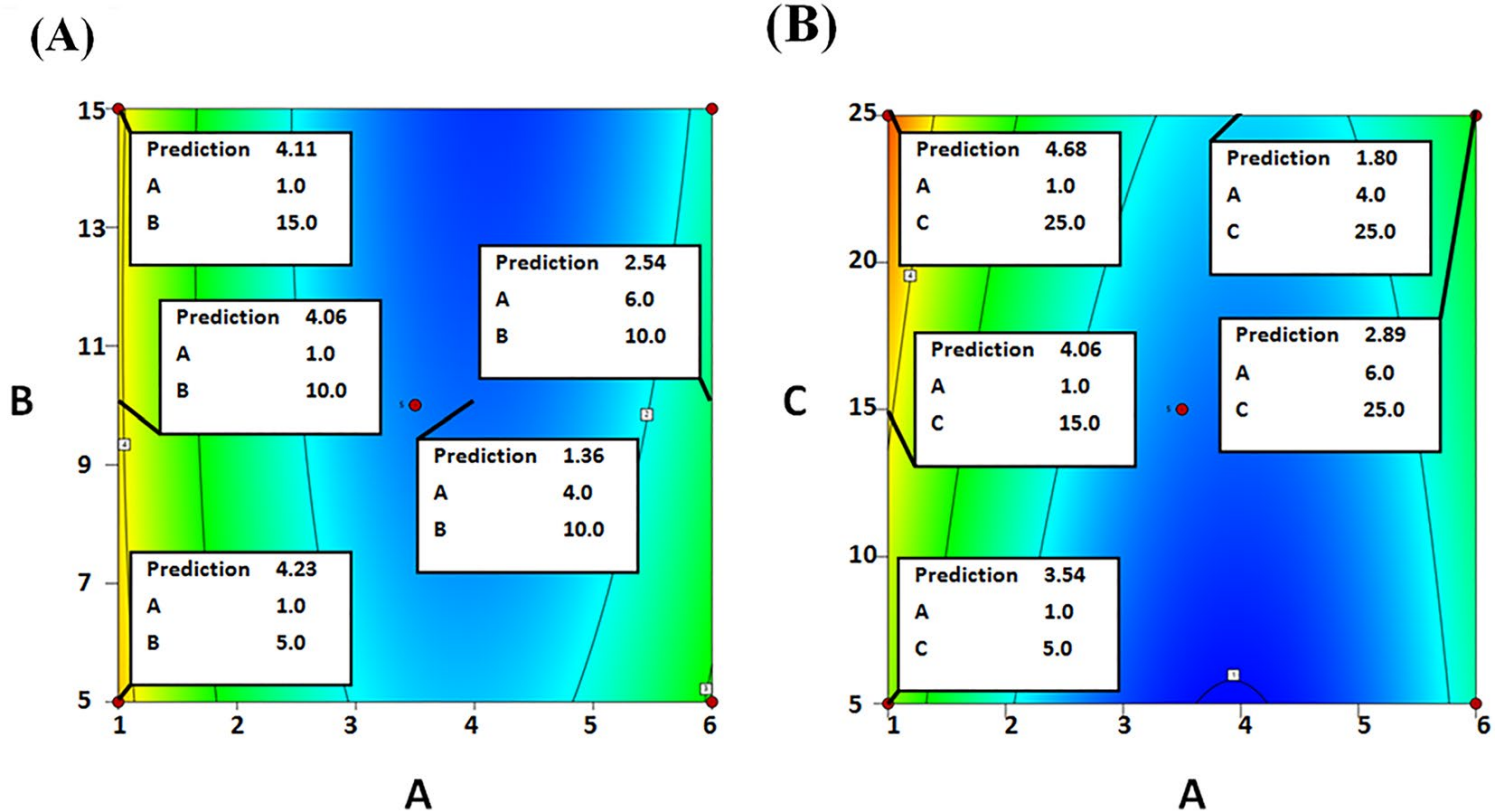
Contour plots exploited to scrutinize the outcomes of varying PP pH (**A**), PP concentration (**B**) and GA concentration (**C**) on the amount of iβ-GL. Two factors were scrutinized in each plot, and the third factor was retained fixed at its zero level.

The data procured after fulfilling the BBD (Table S1) were scrutinized via a quadratic model whose equation was presented as Eq. (1). The P-value and R^2^ of this model were 0.0032 and 0.9271, respectively, and this implied that the model was significant and valid. Table S2 unveiled that the PP pH quadratic and linear terms (A^2^ & A) were the two most significant model terms (0.0001 and 0.0034 P-values, respectively). On the other hand, both the linear and the quadratic terms of PP concentration were insignificant. Noteworthy, when Carr beads were grafted via whey protein isolate, the linear term of WPI concentration was also insignificant whereas the linear term of the WPI pH was significant^22^. Moreover, varying the concentration of the soy protein isolate (SPI), which was utilized to graft Carr beads, elicited only ~ 1.28 fold variation in the amount of iβ-GL whereas varying the SPI pH elicited 2.18 fold variation in the amount of iβ-GL^18^. The intense effect of PP pH could be seen in Fig. 2A which scrutinized the outcomes of varying PP pH (A) and PP concentration (B) whilst retaining GA concentration (C) fixed (central, zero level). Raising the PP pH from 1 to 4 would reduce the amount of iβ-GL from 4.06 to 1.36 Ug^−1^ (− 2.99 fold reduction) if a 10% PP concentration was adopted. Figure 2B, which scrutinized the outcomes of varying PP pH (A) and GA concentration (C) whilst retaining PP concentration (D) fixed (central, zero level) also revealed that raising the PP pH from 1 to 4 would trigger a − 2.60 fold reduction in the amount of iβ-GL if a 25% GA concentration was adopted. Thus, pH 1 was chosen as the optimum PP pH. As for the optimum PP concentration and GA concentration, they were recommended to be 10% (w/w) and 25% (v/v), respectively. Utilizing the aforementioned optimum settings was predicted to enable the acquisition of 4.68 Ug^−1^ iβ-GL. Noteworthy, the aforementioned optimum settings were fulfilled in the BBD (run 6), and 4.97 ± 0.11 Ug^−1^ iβ-GL was attained which was close to the predicted 4.68 Ug^−1^.

In order to rationalize the selection of pH 1 as the optimum PP pH, the charges presented via both Carr and PP were considered. Carr was reported to exhibit a negative zeta-potential at pH 1. This negative zeta-potential could be regarded to the Carr sulfate moieties as their pKa is ~ 2^26^. On the other hand, PP would be cationic as its pI was reported to be 4.5–4.8^3,4^. Thus, an ionic-exchange would be created amid Carr and PP at pH 1 and this would promote the PP-Carr grafting at this pH. Noteworthy, pH 2 (lowest inspected pH) was favored for the preparation of the Carr-PP coacervates which were procured after introducing Carr into a double emulsion stabilized via PP. Complex coacervation also depends on the ionic-exchange amid two biopolymers that exhibit opposite charges^24^.

Other forms of interactions could have also been involved in the PP-Carr grafting at pH 1, such as hydrophobic interactions. PP was shown to exhibit surface hydrophobicity at neutral pH and also after being subjected to acidic (pHs 2 and 4) and alkaline (pHs 10 and 12) pH shifting processes^11^. Thus, it would be expected that PP would present surface hydrophobic residues at pH 1. Furthermore, hydrogen-bonding could also be involved in the interactions amid PP and Carr owing to the abundant amino and hydroxyl residues, respectively. Thus, the combined action of ionic-exchange, hydrophobic interactions, and hydrogen-bonding potentiated the interaction amid PP and Carr at pH 1, and this led to the creation of an intense PP coating around Carr beads. The intense PP coating interacted efficiently with GA, and this eventually permitted the immobilization of a big amount of β-GL. Noteworthy, it was previously debated that ionic-exchange, hydrophobic interactions, and hydrogen-bonding were involved in the interactions amid Carr and soy protein isolate (SPI)^18^ and in the interactions amid the polysaccharide blend, agar-gum-tragacanth, and the amino-rich, polyethyleneimine^27^.

As regards to the sharp decline that was observed in the amount of iβ-GL after raising the PP pH from 1 to 4 (Fig. 2), it could be regarded to the aggregation amid PP entities. PP was reported to exhibit its iso-electric point (pI) at pH 4.5–4.8^3,4^. Thus, increasing the PP pH from 1 to 4 would put PP nearer to its pI. Putting PP nearer to its pI was previously reported to be coupled with an increase in aggregation amid its entities. This aggregation was promoted by the decreased repulsions amid PP entities near pI^3,23^. Increased aggregation amid PP would affect the amount of its exposed charged amine residues^23^. If these exposed charged amine residues were reduced, the ionic-exchange amid PP and the anionic Carr would be impaired, and this would eventually reduce the amount of iβ-GL. Noteworthy, it was formerly argued that the aggregation of PP at its pI negatively affected its foaming capability^1^. Taking into account that hydrogen-bonding, hydrophobic and electrostatic interactions contribute to the foaming capabilities of proteins^1^, it might be inferred that the negative effect of PP aggregation would go beyond the ionic interactions amid PP and Carr. It might also affect the hydrogen-bonding and hydrophobic interactions amid PP and Carr. The aggregated PP entities would interact with each other rather than with the Carr beads. In a somewhat similar situation, increased aggregation amid PP was debated to reduce complex formation amid PP and gum-tragacanth^3^.

It could also be observed that further raising the PP pH from 4 to 6 incremented the amount of iβ-GL. For instance, the amount of iβ-GL would increase from 1.80 to 2.89 Ug^−1^ if the PP pH was raised from 4 to 6 whilst adopting a 25% GA concentration and a fixed PP concentration of 10% (Fig. 2B). Moreover, the amount of iβ-GL would increase from 1.36 to 2.54 Ug^-1^ if PP pH was analogously raised whilst adopting a 10% PP concentration and a fixed GA concentration of 15% (Fig. 2A). Raising the PP pH from 4 to 6 would place PP away from its pI (4.5–4.8^3,4^). Thus, the aggregation amid PP would decrease^3,23^, and this would improve the interactions amid PP and Carr. However, it should be noted that pH 6 is above PP pI, and PP would be chiefly anionic at such pH. Nonetheless, some cationic patches would still be present and available to ionically interact with Carr. Noteworthy, legumin, which is amid the PP globulins that constitute 65–80% of PP construction, exhibits a pI 5–6^1^. Thus, it would present cationic moieties at pH 6. These cationic moieties would constitute the cationic patches that would ionically interact with Carr at pH 6. In a somewhat analogous situation, complex formation amid PP and gum-tragacanth at pH 5.54 was argued to be a consequence of the ionic interactions amid the positively charged patches of PP and the anionic gum-tragacanth^3^. Moreover, the evident interactions amid egg white protein (EWP) and GEG beads at pH 7.6, which was also higher than the 4.8–5 pI of EWP, were argued to be mediated via ionic-exchange amid the EWP cationic patches and the anionic GEG^15^.

Noteworthy, the GA-PP grafting processes of agar (Fig. 1C) and Carr were analogously affected by the alterations in PP pH. In both cases, the topmost amount of iβ-GL was recorded at pH 1 (optimum PP pH). Afterwards, the amount of iβ-GL decreased as the PP pH was raised in the direction of PP pI. Moreover, further raising the PP pH beyond its pI increased the amount of iβ-GL. Such similarity could be regarded to the anionic sulfate moieties which are constituents of both Carr^10^ and agar^17^.

With respect to the PP concentration, it was mentioned earlier that its effect on the acquired amount of iβ-GL was insignificant. Figure 2A revealed that raising the PP concentration from 5 to 10 and then to 15% would induce slight undulations in the amount of iβ-GL from 4.23 to 4.06 and then to 4.11 Ug^−1^, respectively, if a PP pH 1 was adopted. This indicated that, at all inspected concentrations, PP presented ample amounts of nucleophilic amine moieties, and this enabled the proficient interaction amid PP and GA. Noteworthy, a similar protein isolate concentration range (5–20%) was investigated during the GA-soy protein isolate grafting of Carr, and it was unveiled that 10% was the optimal soy protein isolate concentration^18^. This optimal concentration was the same as the 10% PP concentration selected herein. It was debated that utilizing 10% soy protein isolate created dense protein shells amid the Carr beads. These shells helped keep the integrity of the Carr beads and also presented ample amounts of nucleophilic amine moieties for the interaction with GA^18^.

The third inspected factor was the GA concentration, and its linear term (C) was significant with a 0.0414 P-value (Table S2). Raising the GA concentration from 5 to 15 and then to 25% would raise the amount of iβ-GL from 3.54 to 4.06 and then to 4.68 Ug^−1^, respectively, if PP pH 1 was adopted (Fig. 2B). Thus, 25% GA solution was selected for the GA-PP Carr grafting. Analogously, 25% GA was selected for the GA-soy protein isolate grafting of Carr^18^. 25% GA was also exploited during the GA-whey protein isolate Carr grafting^22^. Moreover, 20% GA was optimal for the GA-egg white protein GEG grafting^15^. Hence, it could be deduced that proteins prefer interacting with elevated GA concentrations. Noteworthy, raising GA concentration would raise its degree of polymerization^12^.Thus, 25% GA (+ 1 level, optimal) solutions would comprise more polymeric GA moieties than the 5% GA solutions (− 1 level). These polymeric GA moieties would favor the interaction with the moieties, which exhibit no or only limited steric limitations. Proteins are oriented in water so that their hydrophilic amino-acids are located on the protein surface, in contact with water. On the other hand, the majority of their hydrophobic amino-acids are embedded within the protein core^1^. The surface hydrophilic amino-acids would provide ample amounts of the nucleophilic amine moieties, which are required to react with GA^12^. Moreover, the GA interaction with these surface hydrophilic amino-acids would not involve steric limitations. Thus, polymeric GA moieties could proficiently interact with aqueous protein dispersions, and this could be the reason for the selection of the elevated 25% GA concentration.

### Alteration of β-GL loading units

The acquired iβ-GL activity increased progressively upon increasing the β-GL loading units. Nonetheless, this was accompanied with a progressive decline in the immobilization efficiency (Table 1). That is increasing the β-GL loading units, increased the amount of the GA-PP-Carr bound β-GL moieties. However, the deactivation amid these bound β-GL moieties got more pronounced as their amount increased, and this led to the retainment of progressively lower percents of active β-GL moieties. Noteworthy, when the β-GL units, which were utilized to load the GA-soy protein isolate grafted Carr beads, were increased from 6.78 to 32.76 Ug^−1^, a progressive increase in the acquired iβ-GL activity and a concurrent progressive decline in the immobilization efficiency were also observed^18^. Increasing the amount of bound β-GL moieties could induce protein–protein interactions betwixt them. Moreover, their heightened molecular crowdedness could hamper the conformational alterations, which should be performed by the enzyme in order to execute its catalytic role, and this would deactivate^28^ a portion of the loaded β-GL moieties.

**Table 1:** Outcomes of altering the β-GL loading units.

Loading β-GL (A)	Acquired iβ-GL (B)	Residual β-GL (C)	Efficiency %	Activity recovery %
Ug^−1^	Ug^−1^	Ug^−1^	((B/(A − C) × 100)	(B/A × 100)
11.12	1.96 ± 0.03^a,^*	8.20	67.24	17.64
23.26	3.83 ± 0.17^b^	15.83	51.55	16.48
32.08	5.30 ± 0.03^c^	22.16	50.72	15.67
39.92	6.31 ± 0.18^d^	27.40	50.41	15.81
49.93	7.81 ± 0.07^e^	33.21	46.72	15.64
81.87	11.44 ± 0.34^f^	56.72	45.49	13.97

*Variable low case letters reflected significant difference amid the results.

With respect to the chosen β-GL loading activity, the lowest (11.12 Ug^−1^) loading activity could be chosen despite of acquiring only 1.96 Ug^−1^ as the largest efficiency was recorded (67.24%). Thus, the activity loss amid the loaded β-GL moieties would be limited and this would permit for more economic exploitation of the available β-GL. Moreover, the largest (81.87 Ug^−1^) loading activity might also be chosen as it enabled the acquisition of the topmost amount of iβ-GL per gram GA-PP-Carr beads (Table 1). This would reduce the amount of immobilizer needed to acquire a desired activity, and would ease the handling of the biocatalyst particularly if a large iβ-GL activity was needed. Noteworthy, the immobilization efficiency (45.49%) acquired herein after loading the GA-PP-Carr beads with 81.87 Ug^−1^ was superior to the 38.46% β-GL immobilization efficiency, which was acquired by the optimal GA-whey protein isolate grafted Carr beads. It should also be noted that this 38.46% immobilization efficiency was achieved after loading the optimal GA-whey protein isolate grafted Carr beads with only ~ 6 Ug^−1^ β-GL^22^. This indicated that much inactivation occurred amidst the GA-whey protein isolate grafted Carr iβ-GL moieties even though their β-GL loading activity was much reduced. In the case in hand, lowering the β-GL loading to 11.12 Ug^−1^ enhanced the immobilization efficiency to 67.24% (Table 1). This 67.24% efficiency reflected the limited deactivation that happened amidst the GA-PP-Carr bound β-GL moieties and that a more efficient exploitation of the available β-GL would be provided. Accordingly, the GA-PP-Carr beads could be favored to the GA-whey protein isolate Carr beads. The GA-PP-Carr beads were also finer immobilizers than the GA-soy protein isolate Carr beads. The latter beads procured 3.62 Ug^−1^ and 4.28 Ug^−1^ iβ-GL activities with 40.88% and 43.28% immobilization efficiencies after being loaded with 32.76 Ug^−1^ and 38.55 Ug^−1^ β-GL, respectively^18^. These values were significantly lower than those achieved by the GA-PP-Carr beads after being loaded with the comparable β-GL activities of 32.08 Ug^−1^ and 39.92 Ug^−1^ (Table 1).

### Characterization

#### FTIR analysis

**Figure 3:**
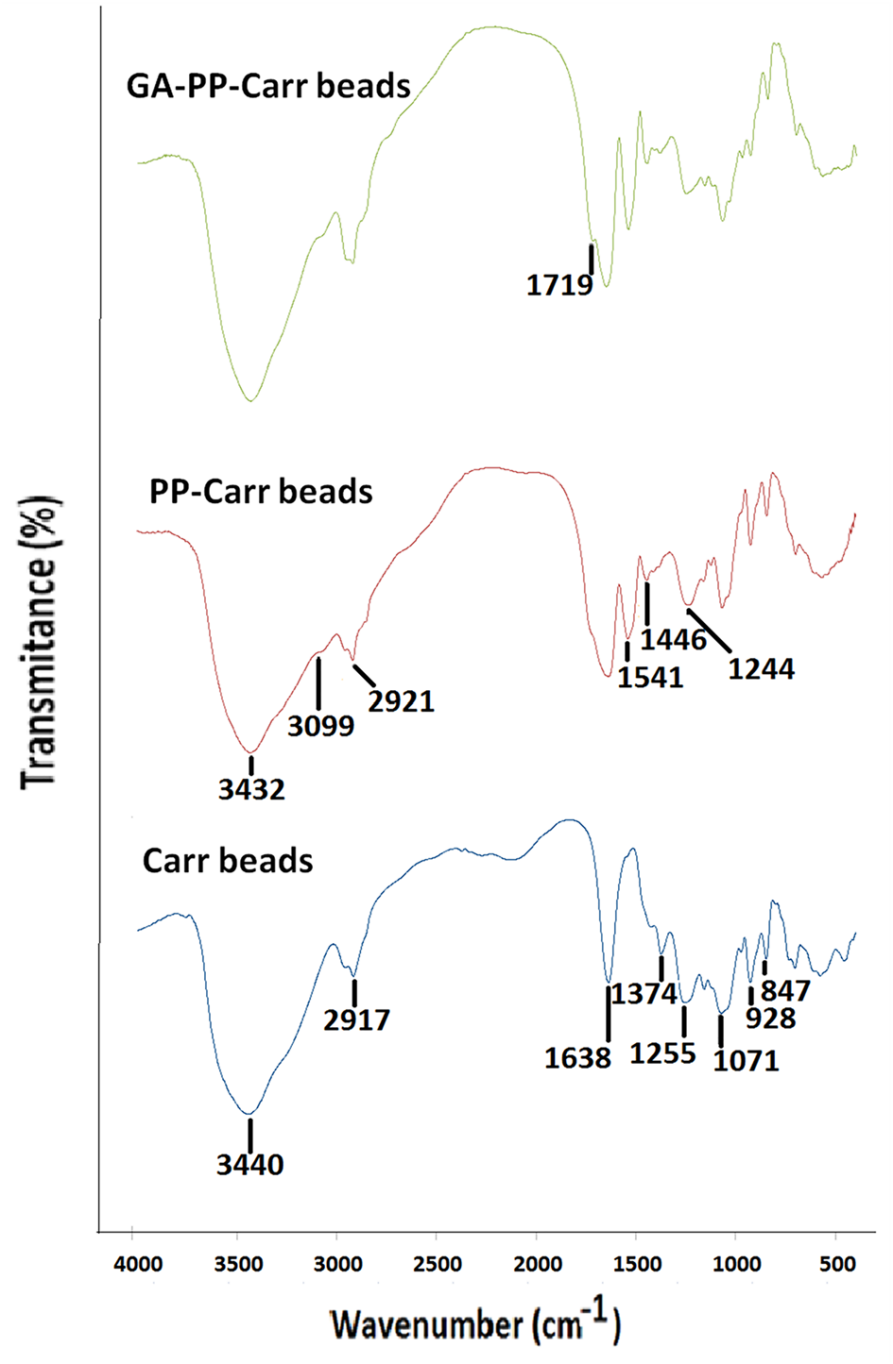
FTIR spectra of Carr beads, optimal PP-Carr beads, and optimal GA-PP-Carr beads.

A robust and broad peak appeared at 3440 cm^−1^ in the FTIR of the Carr specimen (Fig. 3). This peak could be attributed to the stretching vibrations of the plentiful OH groups^29^ of Carr. Moreover, the peak at 2917 cm^−1^ could be attributed to the symmetric and asymmetric stretching vibrations of the CH and CH_2_^29^ in Carr. The Carr FTIR also showed peaks at 1638 and 1374 cm^−1^. Analogous peaks were formerly observed at 1635 and 1368 cm^−1^ in the FTIR of i-carrageenan specimen, and they were attributed to water deformation and C–H stretching, respectively^30^. Noteworthy, Jiang et al.^31^ reported that Carr fingerprint region comprised sulfate ester peak at 1226 cm^−1^, glycosidic bond peak at 1064 cm^−1^, d-3,6-anhydrogalactose peak at 925 cm^−1^ and d-4-sulfate-galactose peak at 843 cm^−1^. The four aforementioned fingerprint peaks appeared herein at 1255, 1071, 928, and 847 cm^−1^, respectively.

As regards to the PP-Carr specimen (Fig. 3), it could be observed that the area of the OH-stretching peak increased and that the peak was slightly perturbed to 3432 cm^−1^. These variations could prove that hydrogen-bonds were involved in the interaction amid PP and Carr as it was formerly debated that the variations in the absorption of OH-stretching reflected variations in hydrogen-bonding^29^. Similarly, the variation in the area of the OH-stretching peak was utilized to prove that hydrogen-bonds were involved in the interaction amid agar-gum-tragacanth blend and polyethyleneimine^27^. Figure 3 also unveiled the appearance of a slight shoulder at 3099 cm^−1^. Moreover, the 2917 cm^−1^ peak in Carr was slightly perturbed to 2921 cm^−1^. Variations in the 3100–2800 cm^−1^ (CH-stretching) region imply that hydrophobic interactions are modified^32^. Thus, the variations recorded herein amid 3099–2921 cm^−1^ could prove that hydrophobic interactions were involved in the interaction amid PP and Carr. It was also observed that Carr sulfate ester peak was perturbed from 1226 cm^−1^, in case of Carr, to 1244 cm^−1^ after interacting with PP. Such perturbing could have occurred secondary to the ion-exchange amid the anionic Carr sulfate residues and the cationic residues of PP. From the above discussion it could be seen that FTIR of the PP-Carr specimen proved that ion-exchange, hydrophobic interactions, and hydrogen-bonding were involved in the interactions amid PP and Carr.

The FTIR of the PP-Carr specimen should also show peaks for the PP amino and carboxylic residues. Nonetheless, the broad OH-stretching peak masked the primary and secondary amino NH-stretching peaks, which were supposed to appear at 3400–3380 + 3345–3325 and 3360–3310 cm^–1^, respectively^33^. Similarly, the NH-stretching peaks, which were supposed to appear after the interaction of agar-gum-tragacanth blend with polyethyleneimine, were masked via the broad OH-stretching peak^27^. As regards to the primary and secondary amino NH-bending peaks (1650–1550 cm^−1^)^33^, they could be represented via the peak which was newly elaborated at 1541 cm^−1^ (Fig. 3). Another peak was elaborated in the PP-Carr FTIR at 1446 cm^−1^. This peak might represent the PP carboxylate residues. These carboxylate residues interacted with the cationic PP amino residues, and this could have caused them to appear at higher wave-number than that regularly reported for carboxylate residues (1420–1300 cm^−1^)^33^. As regards to the GA-PP-Carr specimen, a new shoulder appeared at 1719 cm^−1^. This shoulder could be attributed to the aldehyde moieties (1740–1725 cm^−1^)^33^ of GA.

#### Elemental construction

The Carr beads comprised 46.02 ± 2.62% carbon, 12.19 ± 1.39% oxygen, and 2.59 ± 0.13% sulfur. Bountiful potassium and chloride were also observed in the Carr beads, which could be regarded to the gelling KCl solution. As regards to the PP-Carr beads, they comprised 43.34 ± 3.10% carbon, 45.40 ± 3.07% oxygen, 1.53 ± 0.43% sulfur, and 9.73 ± 0.44% nitrogen. The nitrogen of the PP-Carr beads was solely derived from PP and this confirmed the merger of PP with Carr. Noteworthy, nitrogen appearance was previously exploited to confirm the merger of variable synthetic and natural polyamine compounds with altered polysaccharide matrices, such as the merger of polyethyleneimine with agar-gum-tragacanth disks^27^ and the merger of soy protein isolate with Carr beads^18^. The GA-PP-Carr beads were also scrutinized, and they comprised 56.00 ± 0.95% carbon, 39.71 ± 0.73% oxygen, 0.21 ± 0.06% sulfur, and 4.07 ± 0.50% nitrogen. That is a 1.29 fold raise was observed in the beads carbon percent after interacting with GA. Such a rise in the beads carbon percent could be regarded to GA as it comprised five carbons (C_5_H_8_O_2_). Analogously, 1.15 fold raise was observed in the carbon percent of soy protein isolate grafted Carr beads after interacting with 25% GA^18^.

#### SEM inspection

**Figure 4:**
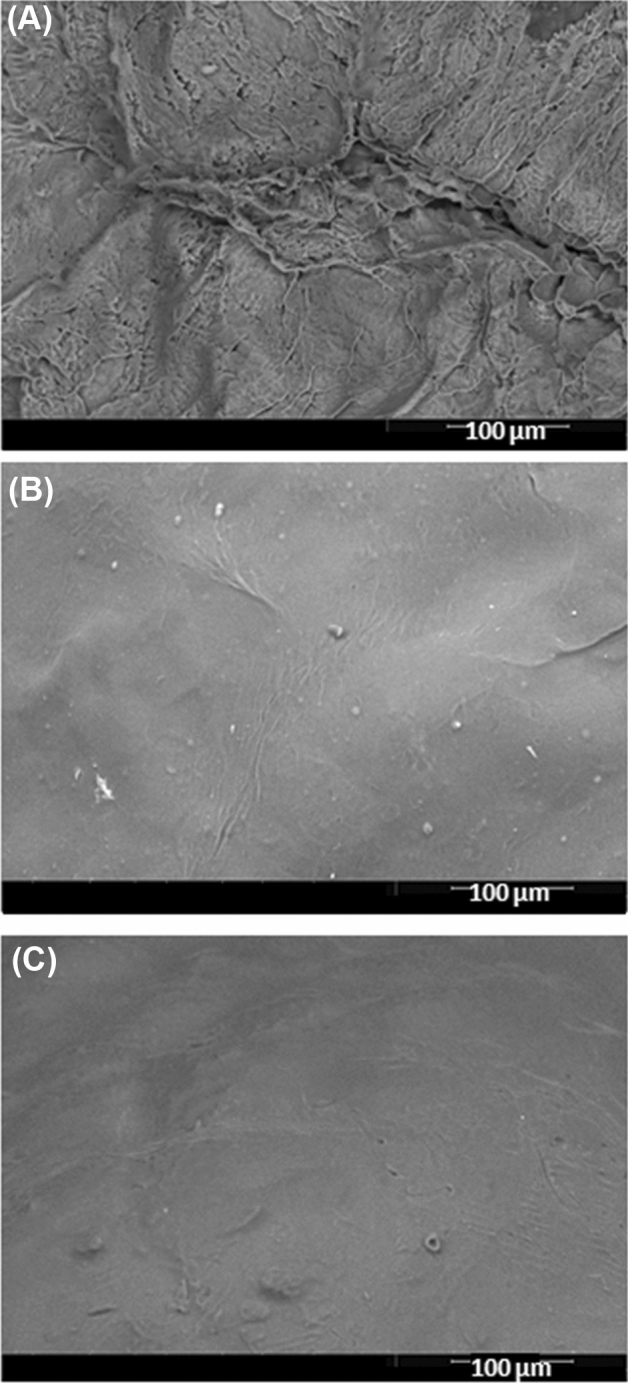
SEM images showing the surfaces of lyophilized (**A**) Carr beads, (**B**) optimal PP-Carr beads, and (**C**) optimal GA-PP-Carr beads at a 800 X magnification.

SEM inspection served to prove the sequential grafting of PP and GA onto the Carr beads. The lyophilized Carr beads surface was irregular with lots of corrugations and evident pores (Fig. 4A). Lyophilization is known to induce pores within hydrogels^34^. Thus, the lyophilized Carr beads irregular porous surface could have been induced by lyophilization. Nonetheless, the beads surface became progressively smooth after the grafting with PP and then GA (Fig. 4B,C). PP created dense protein shell amid the Carr beads. This shell guarded against the surface damaging effects of lyophilization and helped the PP-Carr beads to attain a relatively smooth surface. Cross-linking with GA fortified the PP shell, and this further guarded against the surface damaging effects of lyophilization. Hence, the smoothness of the GA-PP-Carr beads was further enhanced. Similarly, grafting Carr with GA-soy protein isolate and GA-whey protein isolate progressively enhanced the smoothness of the lyophilized beads surfaces^18,22^.

### Temperature profile and reckoning of activation energy

**Figure 5:**
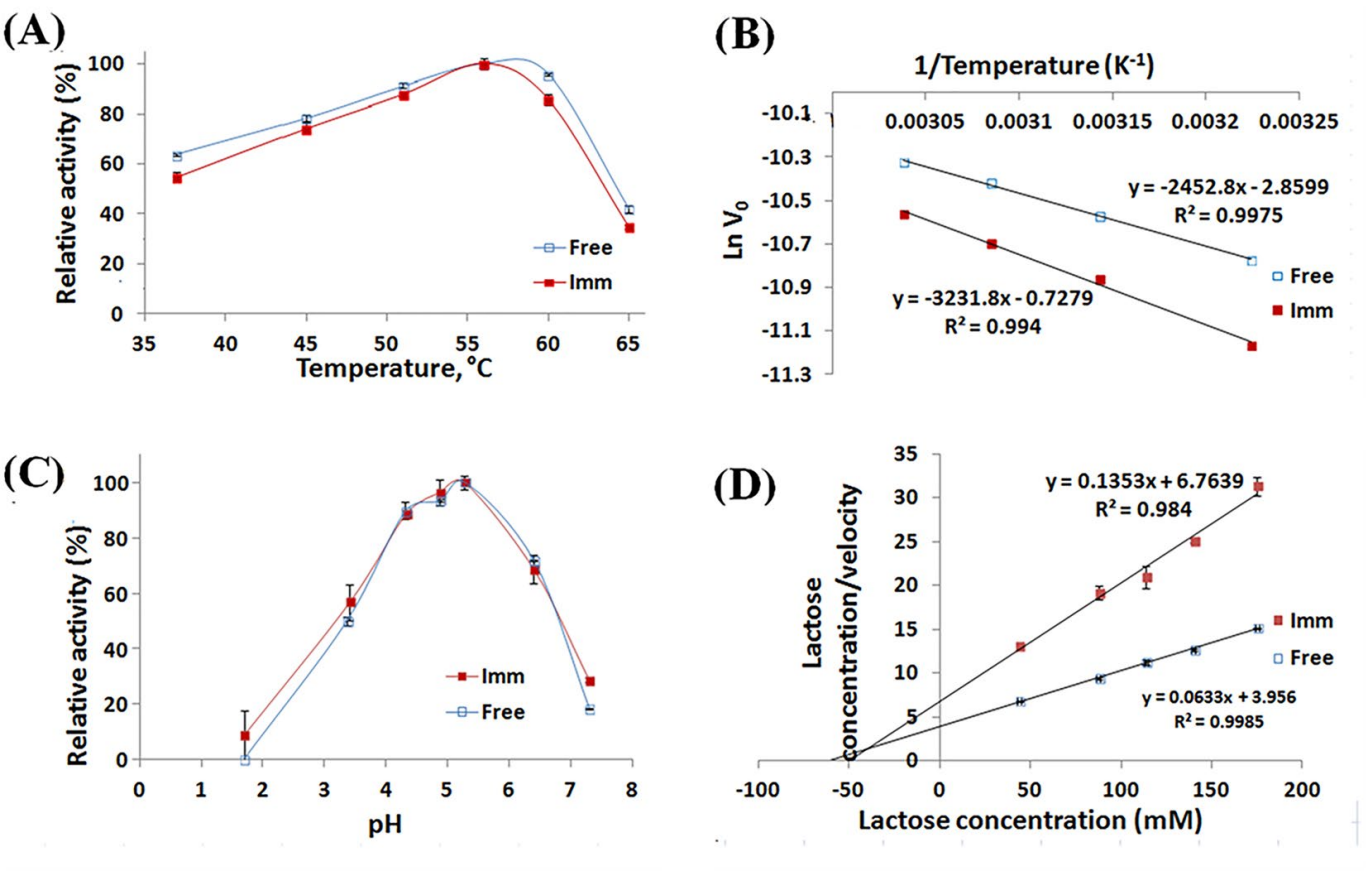
(**A**) Temperature profiles manifested by the free and the GA-PP-Carr iβ-GLs (**B**) Plots of 1/temperature vs Ln intial lactose degradation rate (Ln V_0_) whose slopes (− E_a_/R) was exploited to reckon the E_a_ for both β-GL specimens. (**C**) pH profiles manifested by the free and the GA-PP-Carr iβ-GLs. (**D**) Hanes–Woolf plot exploited to reckon the free and the GA-PP-Carr iβ-GLs K_m_ and V_max_ values.

The free β-GL topmost activity was manifested at 56 °C (Fig. 5A). This was in accordance with the former reports that stated that the free *A. oryzae* β-GL manifested its topmost activity at 55 °C^15,22^. The β-GL temperature optimum was not affected by its immobilization via the GA-PP-Carr beads. Analogously, the *A. oryzae* β-GLs temperature optima were unaltered following the covalent binding to GA-whey protein isolate grafted Carr beads^22^ and the bio-affinity adsorption onto concanavalin A-layered-silica-coated titanium-dioxide^35^. Moreover, the β-glucosidase-Zn_3_(PO_4_)_2_ hybrid nanoflower and its free compeer manifested similar temperature optima^36^. As regards to the activation energy (E_a_) for lactose catalytic degradation, it was reckoned from Fig. 5B slopes. The free β-GL manifested an E_a_ of 20.39 kJ mol^−1^ which was close to the 21.22 kJ mol^−1^ E_a_ formerly recorded for the selfsame enzyme^18^. On the other hand, the GA-PP-Carr iβ-GL manifested a larger E_a_ of 26.87 kJ mol^−1^. Noteworthy, the E_a_ of *Enterobacter aerogenes* β-GL was also enlarged after its entrapment within agar^37^. Furthermore, the E_a_ of protease was enlarged after its encapsulation in poly(vinylimidazole)/clay gel^38^. E_a_ is the energy difference amid the reactants and the transitional entities, which would later decompose into products, and it indicates the extent of the variation that would occur in the reaction rate if the temperature was varied^39^. Thus, the larger E_a_ recorded herein indicated that the iβ-GL manifested bigger variations in its reaction rate upon raising the temperature from 37 to 56 °C. Analogously, it was debated that the larger E_a_ of phenol oxidase reflected its higher sensitivity to temperature rise^39^.

### Inspection of free and GA-PP-Carr iβ-GLs pH profiles

Both free and GA-PP-Carr iβ-GLs manifested their topmost activity at pH 5.3 (Fig. 5C). Nonetheless, the GA-PP-Carr iβ-GL optimum pH range could be extended to pH 4.9 as the activities manifested at pH 4.9 were statistically insignificant (P-value = 0.5253) from those manifested at pH 5.3. Noteworthy, it was previously reported that both the free and the GA-whey protein isolate grafted Carr beads iβ-GLs manifested their topmost activity at the same pH range^22^. Analogous situations were also recorded after β-GL entrapment within agar^37^ and after its covalent binding to tannic acid stabilized silver nano-particles^40^ and to genipin grafted alginate-gelatin^41^.

### Reckoning of K_m_ and V_max_

The free β-GL acquired K_m_ of 62.5 mM, and V_max_ of 15.8 µmol/min mg enzyme (Fig. 5D). Immobilization reduced both of these values, and only 50.0 mM K_m_ and 7.4 µmol/min mg enzyme V_max_ were recorded for the iβ-GL. Noteworthy, both the K_m_ and V_max_ of catalase were reduced after catalase adsorption onto poly(2-hydroxyethyl-methacrylate-glycidyl-methacrylate) cryogel^42^. The K_m_ and V_max_ of *A. oryzae* β-GLs were also reduced after the covalent linking to GA-copper gelled^28^. Moreover, reduction in K_m_ was also observed after the adsorption of *A. oryzae* β-GL onto graphene-iron-oxide nano-composites^43^ and onto concanavalin A-layered-silica-coated titanium-dioxide^35^. The reduction in K_m_ implied that the β-GL affinity for its substrate increased. Such an increased affinity could be regarded to the conformational alterations that occurred in the iβ-GL as these conformational alterations could have promoted the substrate accessibility to the active site^43^. Noteworthy, lactose is the substrate involved in variable β-GL applications, such as whey valorization, preparation of the prebiotic galacto-oligosaccharides, and preparation of low lactose or lactose free dairy products^14^. Thus, the GA-PP-Carr iβ-GL increased affinity to lactose would encourage its exploitation in these industrial applications.

With respect to the drop in V_max_, reductions in V_max_ were formerly observed following the immobilization of variable enzymes, such as β-GL^22,40,44^, catalase^42^, and protease^27^. V_max_ reduction was previously regarded to the diffusional restrictions imposed onto the immobilized enzymes. V_max_ represents the velocity of the enzyme at the point of saturation^45^. If the diffusion of substrates and products to and from the enzyme active site was restricted, the enzyme velocity would be reduced especially at saturation as lots of substrate and product moieties would be present. Noteworthy, V_max_ reduction was also regarded to the immobilization induced enzyme inactivation^46^. Such inactivation would reduce the amount of active enzyme entities, and this would consequently reduce the apparent enzyme velocity.

### Reusability

**Figure 6:**
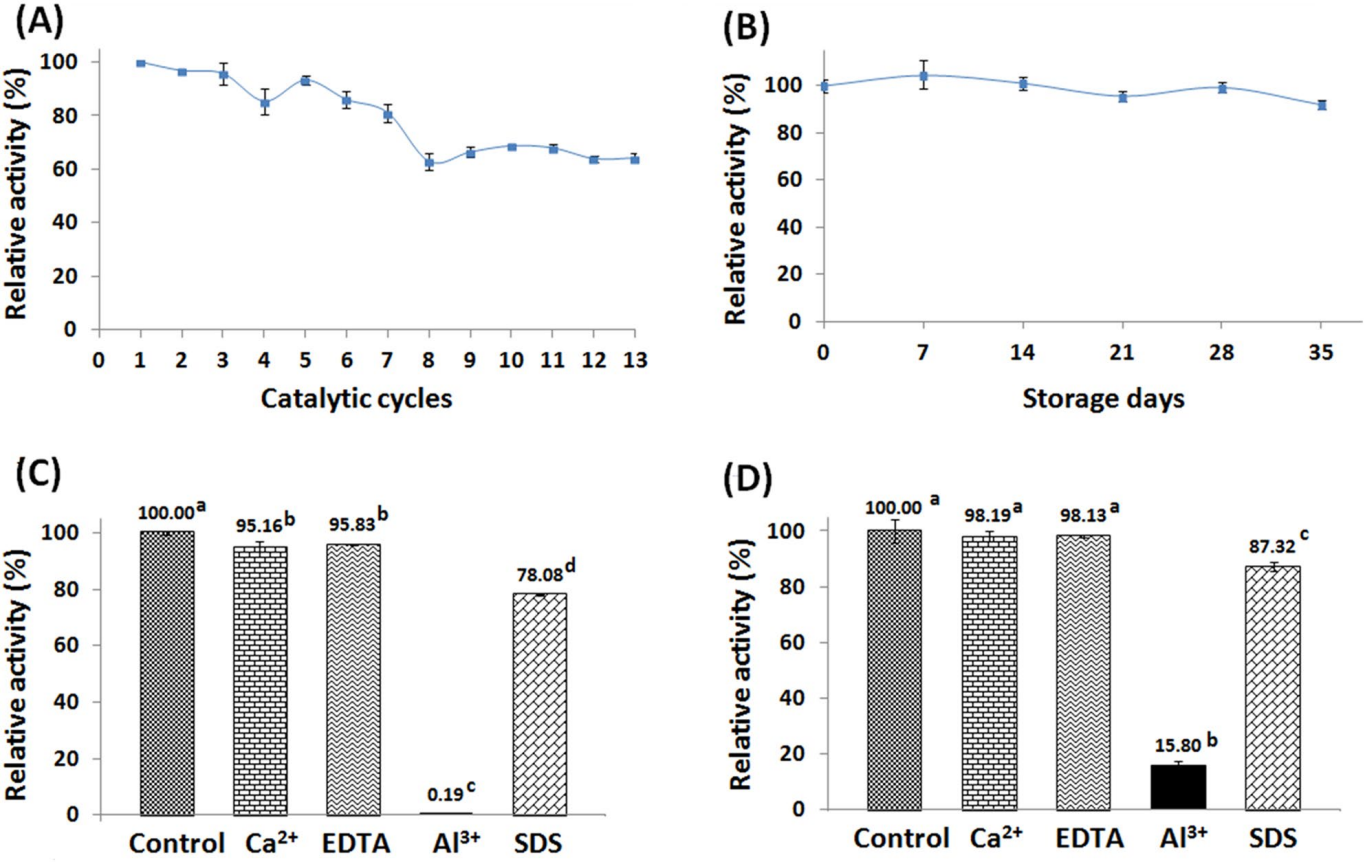
(**A**) operational stability and (**B**) storage stability of the GA-PP-Carr iβ-GL. Stability of the (**C**) the free β-GL and (**D**) its immobilized analogue amid Ca^2+^, Al^3+^, SDS, and EDTA (Variable low case letters reflected significant difference amid the results).

The GA-PP-Carr iβ-GL exhibited gradual reduction in its relative activity during its reuse. Figure 6A revealed that 68.68 ± 0.32% and 64.14 ± 1.6% of its inceptive activity were offered during the 10th and 13th catalytic cycles, respectively. Such operational stability was superior to those offered by the *A. oryzae* β-GLs which were entrapped in polydimethylsiloxane-grafted silica composites or were covalently bound to tannic acid stabilized silver nano-particles as these previously iβ-GLs kept only 59.2% and ~ 60% of their inceptive activities during their 6th and 10th catalytic cycles, respectively^40,47^. On another occasion, the *A. oryzae* β-GL cross-linked aggregates, which were constructed via aldehyde dextran, manifested ~ 60% of their inceptive activity during their 10th catalytic cycle^44^. The reduction in the iβ-GL relative activity during its reuse could have occurred secondary to the gathering of relatively big amounts of glucose and galactose (reaction products) at the active site. Such gathering could have restricted the substrate diffusion^27^. Moreover, glucose and galactose are non-competitive and competitive inhibitors for β-GL, respectively. Thus, their inhibition might have contributed to the obtained reductions. It should also be noted that the 3-D configuration of the β-GL active site could have been distorted following its frequent interaction with the substrate^28^. The observed reductions could have also been induced by the uncontrolled interactions amid the GA-PP-Carr and the iβ-GL. Uncontrolled immobilizer-enzyme interactions could negatively affect the immobilized enzyme stability^8,9^.

### Storage stability

The GA-PP-Carr iβ-GL relative activity varied slightly during its storage where 99.29 ± 2.18 and 91.74 ± 1.85 activities were offered during the 28th and 35th storage days, respectively (Fig. 6B). Such values reflected the incremented storage stability of the GA-PP-Carr iβ-GL which could be attributed to the rigidification of the iβ-GL configuration. Multipoint covalent interactions with an immobilizer could rigidify the enzyme configuration, and this rigidified configuration would be more resistant to distortions^8^, particularly under drastic situations. Noteworthy, the *A. oryzae* β-GLs, which were covalently bound to tannic acid stabilized silver nano-particles and were entrapped within silica composites, kept only 77% and 73.9% activities, respectively, after 30 storage days^40,47^. It was also reported that the agar entrapped *Enterobacter aerogenes* β-GL and the barium alginate bound *Enterobacter cloacae* β-GL kept only 78.09% and 63.72% activities after 40 and 30 storage days, respectively^37^.

### Stability in presence of Ca^2+^, Al^3+^, SDS, and EDTA

The free and the GA-PP-Carr iβ-GLs stabilities were assessed in presence of Ca^2+^, Al^3+^, SDS, and EDTA. β-GLs were reported to be inhibited via Calcium ions^14^. Calcium is the chief milk mineral and it is also present in whey. Two thirds of milk calcium form complexes with casein^48^.Thus, they may not interact with or inhibit β-GL^14^. However, the remaining third of milk calcium is in serum phase. Serum calcium is mainly associated with phosphates and citrates, but it also exists, in small amounts, in the ionic state. The amount of ionic calcium could also be raised upon acidification. The acidification could occur during the acid coagulation of milk^48^. Ionic calcium would interact with β-GL and would inhibit it. Hence, the industrial β-GL should manifest fine stability in presence of Ca^2+^. Figure 6C revealed that the free β-GL was significantly inhibited via Ca^2+^ (P-value < 0.05) whereas no significant alteration was recorded in the activity of the GA-PP-Carr iβ-GL (Fig. 6D). The increased GA-PP-Carr iβ-GL Ca^2+^ stability would encourage its industrial use.

The β-GLs stabilities were also explored in presence of EDTA, a known metal ion chelator^35^. The free β-GL was significantly inhibited via EDTA (P-value < 0.05). The inhibition of the *A. oryzae* β-GL via EDTA was formerly reported, and it was attributed to the metalloenzyme nature of this β-GL^35^. As regards to the GA-PP-Carr iβ-GL, no significant alteration was recorded in its activity following its incubation with EDTA (Fig. 6D). Immobilization could have limited EDTA accessibility to the β-GL bound metal ions^35^. Noteworthy, *A. oryzae* β-GL stability versus EDTA was also enhanced following its binding to concanavalin A-layered-silica-coated titanium-dioxide^35^.

Figure 6C and D revealed that the free and the GA-PP-Carr iβ-GLs were significantly inhibited by SDS and Al^3+^ (P-value < 0.05). SDS is an anionic detergent that has the ability to establish complexes with proteins^35^ whereas Al^3+^ is a food contaminant that was found in infant milk formulae^49^. The free β-GL reserved only 78.08 ± 0.19% and 0.19 ± 0.09% activities in presence of SDS and Al^3+^, respectively. On the other hand, the GA-PP-Carr iβ-GL reserved 87.32 ± 1.55% and 15.80 ± 1.82% activities in presence of SDS and Al^3+^, respectively. Such improved stability would encourage the GA-PP-Carr iβ-GL industrial utilization as it might be subjected to these additives during its industrial use. It is worth mentioning that binding of the *A. oryzae* β-GL to concanavalin A-layered-silica-coated titanium-dioxide was formerly shown to improve its stability in presence of SDS. SDS was debated to induce significant alterations in β-GL active site^35^. Immobilizing β-GL through multipoint covalent attachments would rigidify its configuration, and this rigidified configuration would be more resistant to distortions^8^, such as those induced via SDS. Thus, the GA-PP-Carr iβ-GL was more stable in presence of SDS.

### Enzymatic degradation of WP lactose

**Figure 7:**
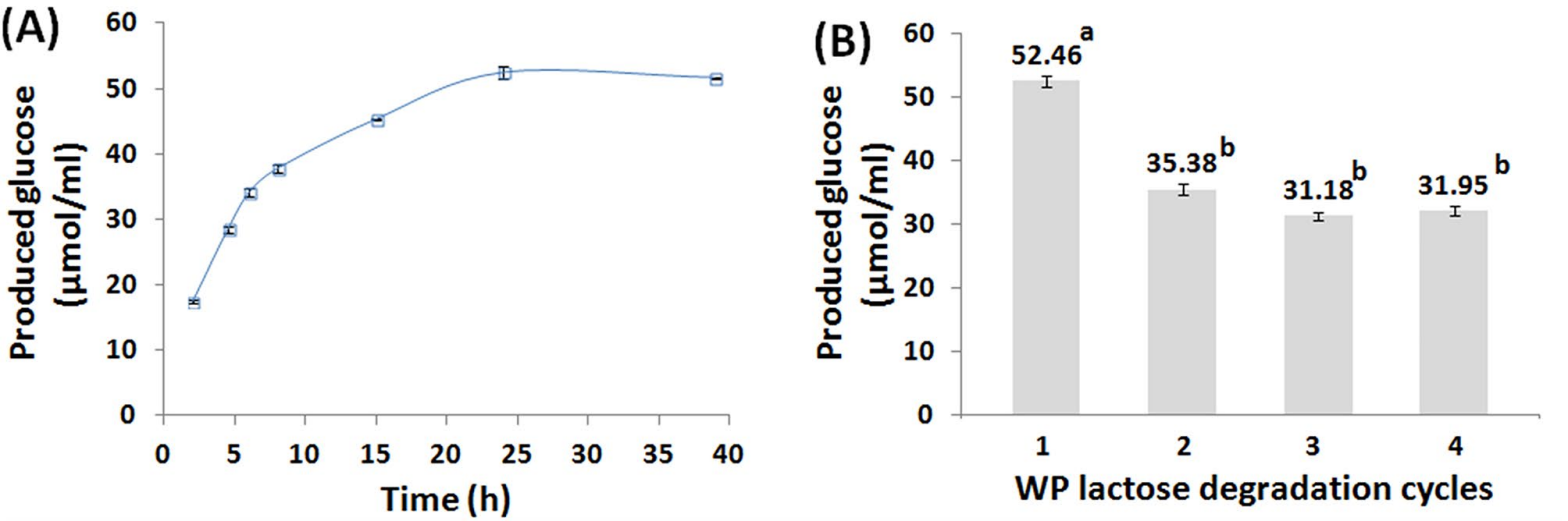
(**A**) Amounts of glucose released from WP after processing it with GA-PP-Carr iβ-GL at 50 °C (**B**) Amounts of glucose released from WP at 24 h after the repetitive exploitation of the selfsame GA-PP-Carr iβ-GL (Variable low case letters reflected significant difference amid the results).

The GA-PP-Carr iβ-GL mediated lactose degradation increased progressively upon extending the degradation time from 2 to 24 h. This was verified from the significant increments recorded in the amount of released glucose (Fig. 7A). Nonetheless, prolonging the degradation time to 39 h didn’t yield any significant variation in the amount of released glucose; thus, 24 h was adopted during the recycling experiment. It should also be noted that the 52.46 ± 0.93 µmol/ml glucose released after 24 h represented 81.90% lactose degradation efficiency (LDE). This efficiency was loftier than the 60.58% and 66.71% LDEs achieved after the degradation of WP and whey lactose via the *A. oryzae* β-GLs which were covalently bound to GA-egg white protein GEG beads^15^ and GA-copper gelled chitosan beads^28^, respectively. Noteworthy, the progressive amassing of glucose and galactose during the prolonged lactose degradation reaction could have prohibited the achievement of whole lactose degradation as glucose and galactose were reported to be non-competitive and competitive inhibitors for β-GL, respectively^28^. Inhibitors were formerly shown to impair enzymes performance. For instance, cellulases are inhibited via phenolics. Accordingly, when no tools were adopted to reduce the phenolics content, 63.5% and 68.2% saccharifiaction yields were obtained from the free and the immobilized Celluclast 1.5L and β-glucosidase cocktails, respectively. On the other hand, when laccase was added to the enzyme cocktails in order to oxidize the phenolics and reduce their content, the saccharification yields were raised to 76.5% and 84.6% for the free and immobilized cocktails, respectively^50^.

The GA-PP-Carr iβ-GL was then recycled for another 3 WP lactose degradation cycles (24 h each). The amount of released glucose dropped significantly during the 2nd cycle and only 35.38 ± 0.88 µmol/ml glucose was released (Fig. 7B). Afterwards, the amount of released glucose undulated insignificantly and 31.95 ± 0.74 µmol/ml glucose was released throughout the 4th cycle. Such glucose amount corresponded to 60.90% of the amount released during the initial lactose degradation cycle. This percent was finer than the 56.55% glucose released during the 4th 24 h whey lactose degradation run which was accomplished via the GA-copper gelled chitosan bound *A. oryzae* β-GL^28^.

## Conclusions

Three covalent immobilizers were concocted after grafting agar, GEG, and Carr with the abundant, cost-effective, PP, and GA. The GA-PP grafted Carr was the most proficient amid the three immobilizers and it was well suited for industrial applications. The GA-PP grafted Carr β-GL immobilization efficiency reached up to 67.24%. Such elevated efficiency would limit the loss of the available β-GL and would make the immobilization protocol more cost-effective. The binding of β-GL to the GA-PP grafted Carr also boosted its affinity for lactose, which is the substrate involved in variable β-GL industrial applications. Moreover, the GA-PP grafted Carr iβ-GL offered good operational stability and superior storage stability where 91.74% activity was offered during the 35th storage day. The stability of the GA-PP grafted Carr iβ-GL was also enhanced amid variable additives, which it might be subjected to during its industrial applications, such as Ca^2+^, EDTA, SDS, and Al^3+^. Finally, the GA-PP grafted Carr iβ-GL was proficient in degrading WP lactose, which is amid the β-GL industrial applications, for four cycles.

## Supplementary Information

Supplementary Tables.

## Data Availability

The author states that the data needed to reproduce the findings of this research are provided within the article.
