# Supplementary material for: Glutaraldehyde-pea protein grafted polysaccharide matrices for functioning as covalent immobilizers
Source: Sci Rep. 2023 Jun 5;13:9105. doi: 10.1038/s41598-023-36045-z (PMC10241775; doi:10.1038/s41598-023-36045-z)
Supplement: Supplementary file 1 — Supplementary Tables. [file 41598_2023_36045_MOESM1_ESM.doc]

| **Run** | **A: PP pH** | **B:PP concentration**  **(%, w/w)** | **C:GA concentration**  **(%, v/v)** | **Iβ-GL activity**  **(Ug^-1^)** |
| --- | --- | --- | --- | --- |
| 1 | 3.5(0) | 15(+1) | 25(+1) | 0.95 |
| 2 | 3.5(0) | 5 (-1) | 25(+1) | 2.39 |
| 3 | 1 (-1) | 10 (0) | 5 (-1) | 3.17 |
| 4 | 3.5(0) | 5 (-1) | 5 (-1) | 1.71 |
| 5 | 6(+1) | 10 (0) | 5 (-1) | 1.97 |
| 6 | 1 (-1) | 10 (0) | 25(+1) | 4.97 |
| 7 | 3.5(0) | 10 (0) | 15 (0) | 1.06 |
| 8 | 6(+1) | 15(+1) | 15 (0) | 2.40 |
| 9 | 3.5(0) | 10 (0) | 15 (0) | 1.39 |
| 10 | 6(+1) | 10 (0) | 25(+1) | 3.25 |
| 11 | 3.5(0) | 15(+1) | 5 (-1) | 1.19 |
| 12 | 3.5(0) | 10 (0) | 15 (0) | 1.84 |
| 13 | 3.5(0) | 10 (0) | 15 (0) | 1.21 |
| 14 | 3.5(0) | 10 (0) | 15 (0) | 1.67 |
| 15 | 1 (-1) | 5 (-1) | 15 (0) | 4.03 |
| 16 | 1 (-1) | 15(+1) | 15 (0) | 4.39 |
| 17 | 6(+1) | 5 (-1) | 15 (0) | 2.80 |

**Table S1:** BBD

**Table S2:** ANOVA of BBD

| **Source** | **SS^a^** | | **DF^b^** | **MS^c^** | **F-value** | **P-value** |
| --- | --- | --- | --- | --- | --- | --- |
| Model | 22.250 | | 9 | 2.470 | 9.890 | 0.0032 |
| A-PP pH | 4.700 | | 1 | 4.700 | 18.800 | 0.0034 |
| B-PP concentration | 0.498 | | 1 | 0.498 | 1.990 | 0.2011 |
| C-GA concentration | 1.550 | | 1 | 1.550 | 6.220 | 0.0414 |
| AB | 0.146 | | 1 | 0.146 | 0.585 | 0.4694 |
| AC | 0.068 | | 1 | 0.068 | 0.272 | 0.6182 |
| BC | 0.213 | | 1 | 0.213 | 0.851 | 0.3868 |
| A^2^ | 14.840 | | 1 | 14.840 | 59.370 | 0.0001 |
| B^2^ | 0.036 | | 1 | 0.036 | 0.145 | 0.7146 |
| C^2^ | 0.004 | | 1 | 0.004 | 0.018 | 0.8977 |
| Residual | 1.750 | | 7 | 0.250 |  |  |
| Lack of Fit | 1.340 | | 3 | 0.445 | 4.310 | 0.0961 |
| Pure Error | 0.414 | | 4 | 0.103 |  |  |
| Cor Total | 24.000 | | 16 |  |  |  |
| ^a^ Sum of squares | |  | | | | |
| ^b^ Degrees of freedom  ^c^ Mean square | |  | | | | |
